# Register of Meteorological Observations

**Published:** 1861-02

**Authors:** 


### Register of Meteorological Observations,

Taken for Atlanta Medical and Surgical Journal, January 1861, at Atlanta, Ga. Latitude 33 deg. 45 min.—Longitude 7 deg. 30 min.—Height above the Sea, 1050 feet.

| Days of Month | BAROMETER. | | | THERMOMETER. | | | RAIN AND SNOW. | | CLOUDS. | | | WINDS. | | |
|---|---|---|---|---|---|---|---|---|---|---|---|---|---|---|
| | 7 A.M. | 2 P.M. | 9 P.M. | 7 A.M. | 2 P.M. | 9 P.M. | Am'unt Rain. | In Sn'w | 7 AM | 2 PM | 9 PM | 7 A.M. | 2 P.M. | 9 P.M. |
| 1 | 29.15 | 29.20 | 29.20 | 35 | 45 | 42 | | | 0 | 5 | 10 | N | N | E |
| 2 | 29.00 | 28.90 | 28.90 | 40 | 42 | 42 | 3.00 | | 10 | 10 | 10 | E | E | S |
| 3 | 28.85 | 28.85 | 28.85 | 42 | 45 | 40 | | | 10 | 0 | 0 | S | S.W. | N |
| 4 | 28.95 | 29.00 | 29.10 | 35 | 42 | 32 | | | 10 | 0 | 0 | N | N | N |
| 5 | 29.15 | 29.20 | 29.25 | 30 | 48 | 42 | | | 5 | 0 | 0 | N | N | N |
| 6 | 29.20 | 29.15 | 29.10 | 40 | 52 | 50 | | | 0 | 0 | 10 | E | S.E. | E |
| 7 | 29.00 | 28.95 | 29.00 | 47 | 52 | 52 | | | 5 | 10 | 10 | E | E | E |
| 8 | 29.05 | 29.05 | 29.00 | 48 | 58 | 50 | | | 5 | 0 | 5 | E | S | S |
| 9 | 28.95 | 28.75 | 28.75 | 48 | 60 | 55 | | | 10 | 5 | 10 | S | E | S |
| 10 | 28.75 | 28.85 | 28.90 | 47 | 47 | 38 | | | 5 | 0 | 0 | N | N | N |
| 11 | 28.95 | 28.95 | 28.95 | 30 | 48 | 45 | | | 0 | 0 | 0 | N | N | N |
| 12 | 28.90 | 28.95 | 28.95 | 45 | 50 | 48 | | | 5 | 0 | 5 | S | S.W. | S.W. |
| 13 | 28.95 | 28.90 | 28.90 | 48 | 50 | 55 | | | 5 | 10 | 10 | E | E | E |
| 14 | 28.90 | 28.90 | 28.85 | 38 | 38 | 38 | | | 10 | 10 | 10 | E | E | E |
| 15 | 28.80 | 28.65 | 28.55 | 40 | 45 | 52 | 3.00 | | 10 | 10 | 10 | E | E | E |
| 16 | 28.75 | 28.85 | 28.95 | 45 | 52 | 45 | | | 0 | 0 | 0 | S | N | N |
| 17 | 28.85 | 28.85 | 28.85 | 44 | 48 | 45 | | | 5 | 5 | 10 | N | N | E |
| 18 | 28.80 | 28.85 | 28.75 | 48 | 48 | 45 | | | 10 | 10 | 5 | E | E | S |
| 19 | 29.00 | 29.00 | 28.95 | 42 | 48 | 45 | | | 0 | 0 | 5 | N | N | N |
| 20 | 29.00 | 29.05 | 29.00 | 40 | 47 | 44 | | | 5 | 0 | 5 | E | S | E |
| 21 | 29.05 | 29.10 | 29.10 | 42 | 42 | 40 | | | 10 | 10 | 5 | E | E | E |
| 22 | 29.20 | 29.15 | 29.15 | 38 | 44 | 40 | | | 0 | 0 | 5 | E | E | E |
| 23 | 29.00 | 28.90 | 28.85 | 40 | 35 | 35 | | | 10 | 10 | 10 | E | E | E |
| 24 | 28.80 | 28.85 | 28.90 | 37 | 40 | 38 | 2.75 | | 10 | 10 | 5 | E | W | W |
| 25 | 28.95 | 28.96 | 28.96 | 36 | 35 | 35 | | | 10 | 10 | 10 | N | E | E |
| 26 | 28.75 | 28.90 | 29.00 | 38 | 38 | 38 | | | 5 | 5 | 0 | S.E. | N | N |
| 27 | 29.05 | 29.05 | 29.10 | 32 | 40 | 38 | | | 0 | 0 | 0 | N | N | N |
| 28 | 29.15 | 29.10 | 29.05 | 34 | 42 | 38 | | | 0 | 0 | 0 | N | N | N |
| 29 | 29.00 | 29.00 | 28.95 | 38 | 44 | 46 | | | 0 | 0 | 0 | S | S | S.W. |
| 30 | 29.00 | 29.05 | 29.05 | 46 | 50 | 46 | | | 0 | 0 | 0 | S.W. | N | W |
| 31 | 29.05 | 29.00 | 29.00 | 40 | 46 | 48 | | | 0 | 10 | 10 | W | S.W. | E |

EXPLANATION OF TABLE:—0 Signifies Entire Clearness—5 Half Cloudy—10 Entire Cloudiness.

